# Developing a weekly patient safety and quality meeting in a medium-sized GI surgical unit in the United Kingdom

**DOI:** 10.1186/1754-9493-8-6

**Published:** 2014-01-24

**Authors:** John Davies, Srinivas Chintapatla, Glenn Miller

**Affiliations:** 1 Department of General Surgery, York Teaching Hospitals Foundation Trust, York, UK

**Keywords:** Patient safety, Clinical audit, Morbidity and mortality meetings, Complications

## Abstract

**Background:**

Morbidity and Mortality (M&M) meetings are advocated as part of good surgical practice, but have been criticised as a method of improving patient outcomes. Several groups have re-designed the format of M&M meetings to improve reporting of complications, near misses and maximise learning points. As a medium sized department of 8 GI surgeons in the UK, we wished to explore and discuss the complications encountered in our clinical practice in more detail than currently available in our monthly M&M/audit meeting, in order to try and improve the quality of care we deliver to our patients. This article describes the practicalities of introducing a weekly meeting and reports on the initial data generated from the patients discussed.

**Methods:**

Four groups of general surgical patients (both elective and acute) are discussed in depth at the weekly meeting- a) patients whose length of in-patient stay is greater than 7 days (as a surrogate marker of a complicated surgical episode), b) unplanned patient readmissions to our hospital (under any specialty) within 30 days of a previous discharge from the GI surgical service, c) all GI surgical inpatient deaths and d) returns to theatre within the same admission (either planned or unplanned).

**Results:**

The initial data generated from the meeting first six months of the meeting are presented e.g.– 302 length of stay greater than 7 days patient episodes (attributable to complications in 26%, normal variant of disease in 59% and social reasons delaying discharge in 15%).

**Conclusions:**

We feel that these weekly meetings can be helpful in addressing both patient safety and quality issues in more depth than the traditional M&M format, as well as being a valuable training resource for both surgical trainees and consultants alike.

## Background

Most surgical trainees, staff grade surgeons and surgical consultants in UK hospitals and abroad will be familiar with morbidity and mortality (M&M) meetings. Although in the USA academic surgical departments are required to hold a weekly M&M meeting to discuss complications and deaths, in the UK and Europe surgical M&M meetings are often held less frequently, on a monthly or bi-monthly basis, often accompanied with clinical audit presentations. Twenty five years ago in the UK, the Royal College of Surgeons of England demanded that every hospital involved in the training of surgeons should hold regular M&M meetings [1]. Today, attendance at surgical M&M meetings is advocated as part of good surgical practice and is highlighted in new UK surgical revalidation processes.

Although few would argue against the ultimate aim of M&M meetings to improve patient care, many surgeons (both junior and senior) will have attended often infrequent meetings where learning opportunities are few and far between, where only a few cases are discussed at each meeting. Sometimes, cases (often deaths) are chosen for discussion in which little could have been done differently to alter the eventual outcome of the patient. Increasing sub-specialisation within surgical specialities (especially within general surgery) can also reduce the relevance of discussion of sub-specialty cases at full departmental M&M meetings. In many institutions there are no specific guidelines for the content and conduct of the meeting, and meetings may create an environment of defensiveness and blame [2].

Acknowledging some of these deficiencies in the traditional M&M process, several groups have re-designed the format of M&M meetings to improve reporting of deaths, complications and near misses to maximise learning points. Introducing “real time” reporting of any suspected adverse incident in a “no-fault” culture as well as a defined route for corrective action resulted in greater numbers of complications and near miss incidents being discussed in US teaching hospital, inevitably providing more material in which to discuss and reflect on practice in an open manner [3]. More recently in the UK, M&M meetings using a more structured approach to reporting and discussing adverse outcomes to provide a framework for discussion was met with enthusiasm by both clinicians and managers alike [4].

As a group of 8 (3 Upper, 5 Lower) gastrointestinal (GI) consultant surgeons who all admit acute and elective patients (with relevant registrar and middle grade support) practising in a medium sized hospital in the UK, we wished to explore and discuss the quality of care delivered to our patients complications encountered in our clinical practice in more detail than currently available in our monthly M&M/audit meeting, in order to try and improve our quality of care through reflective discussion. We proposed to do this by arranging a weekly half day meeting. Rather than focus on self-reporting of adverse incidents and complications in an attempt to reduce any bias, we elected to discuss all patients who fell into 4 arbitrary categories selected by the senior author and head of department, which we hoped would capture the maximum number of complications and potential learning/discussion points in both elective and acute patients.

These categories are-

a) Length of in-patient stays greater than 7 days (as a surrogate marker of a complicated surgical episode).

b) Unplanned patient readmission to our hospital (under any specialty) within 30 days of a previous discharge from the GI surgical service.

c) All GI surgical inpatient deaths.

d) Returns to theatre within the same admission (either planned or unplanned).

This article describes the practicalities of introducing a weekly meeting and reports on the initial data generated from the patients discussed.

## Methods

After an initial discussion and approval by the Chief Executive and Medical Director about introducing the safety meeting into the GI surgical service with implications upon clinical activity, several matters needed attention. It was agreed that, for a trial period, preparation for, and the time spent in the meeting would take up one consultant half day activity session. Friday morning was chosen as the best time to introduce the session (as at our hospital minimal major theatre activity is undertaken on a Friday morning) which was named the Patient Safety and Quality Meeting. Several changes to the consultant timetable (with regards to day case operation lists and out-patient clinics) were undertaken to free up all the GI consultant surgeons and middle grade (associate specialist, specialist registrar, and staff grade) surgeons. Secretarial and IT support was provided from the departmental budget.

Our hospital (York, UK) uses a novel in-house electronic core patient database system (CPD) which integrates demographic data and all clinical events (such as admission details, theatre records, all investigations and clinic letters) onto a permanent electronic record for each patient. Collaborating with our IT colleagues, this database is interrogated weekly to provide data of the patients for the meeting. The names of the patients to be discussed are emailed to the GI surgical consultants 7 days prior to the meeting. The meeting itself is held in one of our conference rooms, with full IT and electronic radiology access, and attended by all of the GI surgical consultants and the middle grade surgeons on duty. An anaesthetist with intensive care commitments also attends, to provide insight into case discussion from a critical care point of view. The junior surgical grade doctors are also encouraged to attend after they have finished their ward duties, if possible.

Either the patient’s consultant or the middle grade surgeon (if the patient’s consultant is not there) presents each case, using pre-printed data capture sheets designed in house for each of the four categories. Additional file 1 is an example of one of the pre-printed data capture sheets (Readmission within 30 days). After presenting the case, it is opened up for discussion in a constructively critical, non-judgemental reflective style by the whole group, attempting to incorporate some error management strategies from the aviation industry such as the consideration of intuitional, organisational and interpersonal communication factors as well as errors of judgement [5,6]. In order to discourage brevity or complacency with regards to the discussion, we actively encourage forthright discussion and probing questions about the case from anyone attending the meeting, both junior and senior. For each particular category, aside from the general discussion, there are certain mandatory sections on the pre-printed data capture sheets which are discussed and recorded, e.g. in the case of readmission whether the discharge was appropriate and whether the readmission was due to a complication or a new unrelated episode, or in the case of a death, ascertaining whether it was expected or unexpected, and whether senior decision making (middle grade or consultant) took place prior to death. In discussion of readmissions, the consultant who the patient was initially admitted under presents the case. Particular emphasis in the meeting is placed on whether anyone in the group would do anything differently if placed in a similar situation.

After discussion, the cases (and any complications) are prospectively recorded on the datasheet and then onto a database. Aside from discussion of patients in the above four categories, we also encourage discussion about other cases (particularly “near misses”) not previously discussed in the 4 categories, requested by any member of the group. The meeting takes about 2 hours, after which there is usually have a journal club, which often has a safety focus- .e.g. presenting a paper on the human and cognitive factors associated with laparoscopic common bile duct (CBD) injuries [7] after discussing one of our own cases of CBD injury, where a patient was re-admitted after five days with a pin hole diathermy burn in the CBD. As an entire department of General Surgery and Urology (which includes Vascular Surgery), we still have monthly formal audit/M&M meetings, but use these more for educational and audit purposes. The study and dissemination of findings of the study was approved by the Clinical Audit and Effectiveness Team at York Teaching Hospital NHS Foundation Trust.

## Results

Table 1 illustrates the total numbers of patients/patient episodes discussed with a selected summary of relevant clinical data over the 6 months since starting the meeting.

**Table 1:** Numbers of patient episodes/patients discussed and outcomes of the meeting

**Length of stay > 7 days (n = 302 patient episodes) attributable to-**				
Complications 26%		Normal variant of disease 59%	Social reasons delaying discharge 15%	
**Unplanned readmission to our hospital (under any specialty) within 30 days of a previous discharge from the GI surgical service (n = 282 patient episodes)-**				
n = 282	Readmitted to GI Surgery 74%, Readmitted to other hospital specialties 26%			
n = 282	Complications 19%	Ongoing symptoms 39%	Inadequate discharge arrangements <1%	Unrelated separate episode 42%
**Patient deaths in which there was a consultant GI surgical involvement (n = 48 patients)-**				
n = 48	Elective patients 8%		Acute patients 92%	
n = 48	Operated upon prior to death 38%		Not operated upon prior to death 62%	
n = 48	Expected deaths 65%		Unexpected deaths 35%	
n = 48	Senior decision making 98%		No senior decision making 2%	
**Returns to theatre after initial operation within the same admission (n = 32 patients)-**				
n = 32	At least one unplanned return to theatre within same admission (24 patients)		Planned returns to theatre- decision made at initial operation (8 patients)	
	Reasons- anastomotic leak (7), bleeding (3), full thickness wound dehiscence (2), Non-anastomotic infarcted bowel (2), operative abscess drainage (2), small bowel obstruction (2), other (6)		Reasons- Planned re-look laparotomies (4), planned pre-discharge ERCP (1), planned re-endoscopic dilatation (1), insertion of CVP line in theatre (1), planned EUA of abscess (1)	

## Discussion

Our new-style weekly meeting is not unique, but is novel compared to the practice of the majority of GI surgical units in the UK. In discussing patients in the 4 categories outlined above we hope to capture the majority of major complications for discussion within our unit in the aim of improving patient care. By using other surrogate markers of adverse incidents and quality of care (such as returns to theatre, previously demonstrated as a surrogate for surgical quality [8]) and readmission rates not covered in traditional M&M meeting formats we believe our meeting gathers a more robust weekly picture of overall quality of surgical care provided to our patients within our unit. We believe openness and non-critical attitudes (which are difficult to define and measure), driven by supportive departmental leadership are crucial in the success of our weekly meeting, a factor noted by others [3].

We acknowledge that unlike other innovations [3,4] upon the traditional M&M meeting we have not yet incorporated robust corrective action protocols and this remains a future challenge after taking our first step of introducing a weekly patient safety and quality meeting. Furthermore, it must be noted that the weekly meeting takes up valuable time and resources (as opposed to monthly or bi-monthly formats) and ongoing support from senior hospital management is essential in this regard.

As the meeting has evolved over the past 9 months, we have made some changes. We have added in a standardised list of complications (with definitions of major complications, (in a similar fashion to other groups) to the pre-printed data sheets to assist in recording the data and also in the discussion. Due to the sensitivity of our IT system, patients returning for planned elective procedures such as GI endoscopies appear on our lists as readmission cases, and we are working on ways to alter this in the data generation for the meeting. However, in general, we are keen to “over capture” data and be able to dismiss the case as an administration error, rather than miss a potential complication. We are also refining our system to allow us to look at unplanned admissions to hospital (to any medical specialty) post GI endoscopy (these admissions do not routinely appear on our data search as these patients are not formally admitted to hospital for their endoscopic procedure) as part of our endoscopy governance. We are also investigating how to link our discussion of post-operative complications of cancer patients directly into cancer registries.

Although the focus of this article is on the process of starting and developing a weekly patient safety and quality meeting, on initial observation of the collective data (Table 1), our readmission rate (approximately 40 patients per month) appears to be quite high, but from the data generated from our meeting, that it appears that approximately 40% of readmissions to our trust are re-admitted with problems we have classified as a new unrelated separate episode (as opposed to ongoing symptoms, which we have recorded as a separate reason for readmission). It is also of note that the majority of patients who died under our care (62%) were not actually operated upon in their final admission. On a more practical point, it is interesting to note that the GI consultant body felt that the reason approximately 15% of our patients remained under our care for over 7 days due to social reasons. In times of fiscal austerity in state care sectors, this figure may well increase in the future. Improving access to intermediate care could significantly improve patient flow and potentially reduce duration of patient stay times.

## Conclusions

As a group of surgeons, we have found these meetings to be very helpful, allowing the opportunity to spend time discussing cases and complications in more detail than in a traditional M&M format with senior colleagues in a reflective, constructive style. We also feel the meeting strengthens non-technical skills and professional relationships within our unit and dovetails with the internationally increasingly important clinical focus on patient safety [9,10]. We feel these weekly meetings are important in addressing both patient safety and quality issues, as well as being a valuable training resource for both surgical trainees and consultants alike. Since starting the meeting, other surgical specialties within our hospital have established similar meetings in a modified format to suit their own particular needs. In terms of revalidation of practice, we feel that the prospectively collected data generated by these weekly meetings provides good evidence of reflective practice, which is validated by the surgical team almost in real time.

## Competing interests

No external funding was received for this study. The authors declare that they have no competing interests. The study was approved by the Clinical Audit and Effectiveness Team at York Teaching Hospital NHS Foundation Trust.

## Authors’ contributions

GM conceived the idea to introduce the weekly meeting, SC contributed to the format and design of the meeting and JD collected the data and wrote the manuscript. All three authors (JD, SC and GM) contributed to the manuscript. All authors read and approved the final manuscript.

## Supplementary Material

Additional file 1 Data collection sheet. Click here for file

## References

[B1] Campbell WB Surgical morbidity and mortality meetings Ann R Coll Surg Engl 1988 70 363 365 3207327 PMC2498614

[B2] Abdulrasheed I Zira DI Eneye AM Modification of the surgical morbidity and mortality meetings as a tool to improve patient safety Oman Med J 2011 26 290 292 22043440 10.5001/omj.2011.72 PMC3191702

[B3] Stahel PF Flierl MA Smith WR Morgan SJ Victoroff MS Clarke TJ Sabel AL Mehler PS Disclosure and reporting of surgical complications: a double-edged sword? Am J Med Qua 2010 25 398 401 10.1177/1062860610370989 20592238

[B4] Higginson J Walters R Fulop N Morbidity and mortality meetings: an untapped resource for improving the governance of patient safety? BMJ Quality and Safety 2012 21 576 585 10.1136/bmjqs-2011-000603 22556308 PMC3382446

[B5] Helmreich RL Education and debate: on error management: lessons from aviation BMJ 2000 320 781 785 10.1136/bmj.320.7237.781 10720367 PMC1117774

[B6] Clarke DL Furlong H Laing GL Aldous C Thomson SR Using a structured morbidity and mortality meeting to understand the contribution of human error to adverse surgical events in a South African regional hospital S Afr J Surg 2013 51 122 126 10.7196/sajs.1537 24209695

[B7] Way LW Stewart L Gantert W Liu K Lee CM Whang K Hunter JG Causes and prevention of laparoscopic bile duct injuries: analysis of 252 cases from a human factors and cognitive psychology perspective Ann Surg 2003 237 460 469 12677139 10.1097/01.SLA.0000060680.92690.E9 PMC1514483

[B8] Morris AM Baldwin LM Matthews B Dominitz JA Barlow WE Dobie SA Billingsley KG Reoperation as a quality indicator in colorectal surgery: a population-based analysis Ann Surg 2007 245 73 79 10.1097/01.sla.0000231797.37743.9f 17197968 PMC1867944

[B9] McCafferty MH Polk HC Patient safety and quality in surgery Surg Clin North Am 2007 87 867 881 10.1016/j.suc.2007.06.001 17888785

[B10] Stahel PF Clavien PA Hahnloser D Smith WR A new journal devoted to patient safety in surgery: the time is now! Patient Saf Surg 2007 1 1 10.1186/1754-9493-1-1 18271986 PMC2222679

