# Supplementary material for: Developing a weekly patient safety and quality meeting in a medium-sized GI surgical unit in the United Kingdom
Source: Patient Saf Surg. 2014 Jan 24;8:6. doi: 10.1186/1754-9493-8-6 (PMC3904932; doi:10.1186/1754-9493-8-6)
Supplement: Additional file 1 — Data collection sheet. [file 1754-9493-8-6-S1.doc]

**Additional file 1- Data collection sheet**

**READMISSION UNDER ANY SPECIALTY WITHIN 30 DAYS**

Consultant Date of discussion ……………

| Patient details | Age  Sex |
| --- | --- |

| CASE SUMMARY: |
| --- |

| Date of previous discharge  Self-discharge  Primary Surgery elsewhere | Y / N  Y / N | |
| --- | --- | --- |
| Service discharged from | GS  Urol  MFE  GM  Other: | |
| Readmitted to: | GS  Urol  MFE  GM  Other: | |
| Date of re-admission |  | |
| Related to original admission | Y / N | |
| Appropriate re-admission | Y / N (e.g. ward attender) | |
| Previous discharge appropriate | Y / N | |
| Explanation |  | Complication  Inadequate discharge arrangements  Unresolved issue (i.e. ongoing symptoms)  Separate Episode |
|  |
|  |
